# GABA_A_ Receptors Are Involved in the Seizure Blockage Prompted by a Polyphenol-Rich Extract of White Grape Juice in Rodents

**DOI:** 10.3390/ph18020186

**Published:** 2025-01-30

**Authors:** Alessandro Maugeri, Rita Citraro, Antonio Leo, Caterina Russo, Michele Navarra, Giovambattista De Sarro

**Affiliations:** 1 Department of Veterinary Sciences, University of Messina, I-98168 Messina, Italy; amaugeri@unime.it; 2 Department of Science of Health, School of Medicine and Surgery, University “Magna Græcia” of Catanzaro, I-88100 Catanzaro, Italy; citraro@unicz.it (R.C.); aleo@unicz.it (A.L.); desarro@unicz.it (G.D.S.); 3 Department of Chemical, Biological, Pharmaceutical and Environmental Sciences, University of Messina, I-98168 Messina, Italy; caterina.russo1@unime.it

**Keywords:** grape juice, *Vitis vinifera*, polyphenols, seizures, epilepsy, natural products, molecular docking

## Abstract

**Background/Objectives:** Polyphenols have been suggested to possess anticonvulsant properties, which can be exploited as tools in novel strategies against epilepsy. Along that line, the aim of this study was to investigate the effects of a polyphenol-rich extract of white grape juice (WGJe) in different rodent models of epilepsy, exploring its putative mechanism of action. **Methods**: In this study, we employed pentylenetetrazole (PTZ)-injected ICR-CD1 mice, audiogenic seizure (AGS)-susceptible DBA/2 mice and WAG/Rij rats. Seizures were monitored and scored, while absence was assessed by electroencephalogram. The open-field test was employed to assess the anxiolytic effects of WGJe. In order to assess the involvement of the GABA_A_ receptor, we used the antagonist flumazenil in AGS-susceptible DBA/2 mice. Computational analyses were employed to evaluate the interaction of the main polyphenols of WGJe and GABA_A_ receptors. **Results**: Our results showed that the intraperitoneal injection of WGJe hindered tonic seizures in PTZ-injected ICR-CD1 mice. In WAG/Rij rats, WGJe did not elicit any significant effects on spike-wave discharges compared to untreated rats. In AGS-susceptible DBA/2 mice, WGJe significantly hampered both clonic and tonic seizures, as well as induced anxiolytic effects. Interestingly, when administering WGJe with flumazenil to DBA/2 mice, we noted that the observed effects were mediated by the GABA_A_ receptor. Moreover, docking simulations confirmed that the main polyphenols of WGJe are able to interact with the benzodiazepine sites located in both extracellular and transmembrane domains in the GABA_A_ receptor. **Conclusions:** This study outlines the mechanism underlying the anti-epileptic activity of WGJe, thus supporting its potential role in the management of epilepsy.

## 1. Introduction

According to the last definition proposed by the International League Against Epilepsy (ILAE), epileptic syndrome is a characteristic cluster of clinical and electroencephalogram features, which is often supported by specific etiological findings (i.e., structural, genetic, metabolic, immune and infectious). Moreover, diagnosis frequently carries prognostic and treatment implications, since epileptic syndromes often have age-dependent manifestations and a range of specific comorbidities [1]. Epilepsy affects as many as 70 million individuals globally and has an incidence of 50/100,000 patients per year and a prevalence of 700/100,000, accounting for more than 0.5% of the total worldwide illness burden [2]. The main strategy for treating the majority of cases is the employment of drugs, both in monotherapy as well as in combination, for drug-resistant conditions [3]. Antiseizure drugs (ASDs) were introduced into clinical practice several decades ago and their development is unceasing. Indeed, the first generation of ASDs started with phenobarbital, which was followed by many other different molecules. In the late 1980s, the second generation started with vigabatrin and continued until the early 2000 with the approval of pregabalin, which set off the third generation of ASDs [3]. Currently, the last approved ASDs by FDA in the USA and EMA in Europe are cenobamate for focal seizures in adults and fenfluramine for seizures in Dravet syndrome (DS) [4]. In addition to synthetic ASDs, natural products have been claimed to possess anti-epileptic potential. The most relevant case is that of cannabidiol, which was approved for treatment in 2018 [5]. It is characterized by a complex mechanism of action, including modulation of neurotransmitters (i.e., glutamate and γ-aminobutyric acid—GABA), intracellular proteins (i.e., phosphatidylinositol 3-kinase) and the endocannabinoid system [6]. The multi-target capability of cannabidiol also applies to other natural products, forming the characteristic of these products and the element exploitable in therapy, especially in resistant forms of epilepsy [7]. From a pre-clinical point of view, many studies have been conducted on polyphenols in relation to diseases affecting the central nervous system, among which is epilepsy [8].

The health-promoting properties of white grape (*Vitis vinifera* L.) are widely acknowledged, which are due to the presence of different active compounds, mainly polyphenols [9]. Along this line, we previously demonstrated that a polyphenol-rich extract of white grape juice (WGJe) was able, in vitro, to reduce both drug-induced toxicity [10] and microbial proliferation [11], whereas, in vivo, it elicited neuroprotective effects in a mouse model of multiple sclerosis [12] and reduced fat accumulation in obese zebrafish [13].

Based on these premises, we assessed the potential anticonvulsant effects of WGJe in different rodent models of epilepsy, investigating the putative mechanism of action.

## 2. Results

### 2.1. Polyphenolic Profile of WGJe

As previously described [10,12], the quali-quantitative composition of WGJe determined by UPLC/QqQ–MS/MS analysis contained several polyphenolic classes, among which flavonols, flavanols, hydroxycinnamates and resveratrols (Table 1). In detail, the most abundant compounds (more than 2 g/kg of WGJe) were quercetin-3-glucuronide, quercetin-3-glucoside, procyanidin B1 and B3, catechin, trans-coutaric acid and cis-piceid.

### 2.2. Effects of WGJe Treatment on Pentylenetetrazole (PTZ)-Induced Seizures

**Figure 1:**
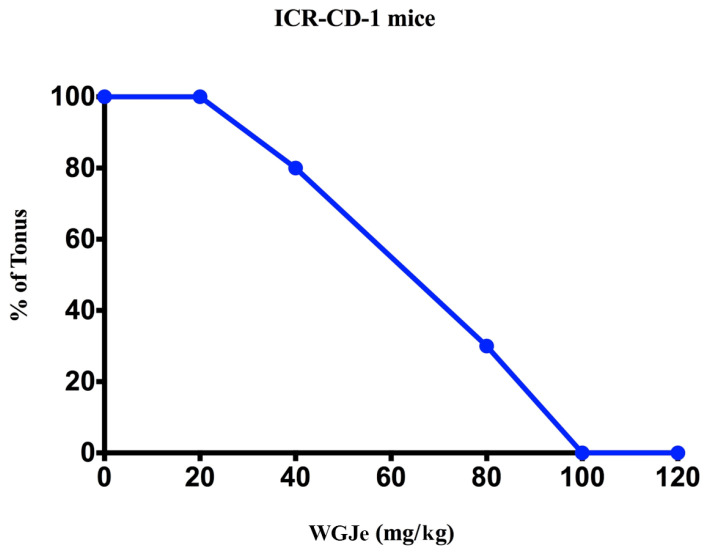
Dose–response curve for the anticonvulsant effect of WGJe (20–120 mg/kg; i.p.) in ICR-CD1 mice intraperitoneally treated with PTZ (60 mg/kg). Abscissa shows the drug doses, ordinate shows the % of tonic seizures induced by PTZ.

Mice pre-treated with the vehicle, before pentylenetetrazole (PTZ) injection, an inhibitor of the GABA_A_ receptor, showed both clonic and tonic seizures, and 80% died within 30 min. WGJe (20 mg/kg intraperitoneally—i.p.), administered 30, 60 and 120 min before PTZ injection, did not significantly (*p* > 0.05) change the incidence of either tonic or clonic seizures. At odds, the treatment with WGJe from 40 mg/kg to 120 mg/kg, 30 min before PTZ injection, produced significant (*p* < 0.01) dose-dependent protection against tonic seizures (Figure 1) with a ED_50_ value of 57.76 (45.48–73.36) mg/kg (Table 2). However, WGJe did not significantly (*p* > 0.05) influence the incidence of clonic seizures. Similarly, WGJe at the doses of 40, 80, 100 and 120 mg/kg, administered 60 and 120 min before PTZ, was not effective against clonic and tonic seizures.

### 2.3. Effects of WGJe on Absence Seizures in WAG/Rij Rats

All WAG/Rij rats, at 6 months of age, exhibited spontaneously occurring spike-wave discharges (SWDs) on electroencephalograms (EEGs). The mean number of SWDs (nSWDs) for a 30 min epoch was 6.78 ± 1.18 seizures, with a mean total duration (dSWDs) of 21.5 ± 4.24 s and a mean single duration (sSWD) of 3.17 ± 0.8 s. The i.p. administration of WGJe in WAG/Rij rats, at the doses reported above, did not significantly (*p* > 0.05) modify the nSWDs and/or dSWDs in comparison to untreated rats.

### 2.4. Effects of WGJe on Audiogenic Seizure (AGS)-Prone DBA/2 Mice

**Figure 2:**
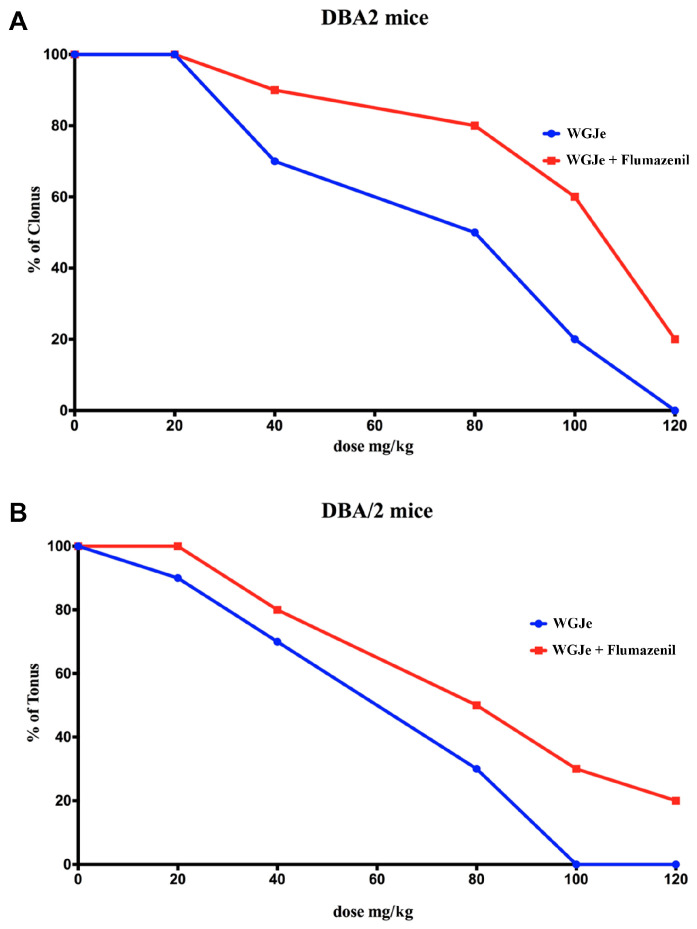
Dose–response curves for the anticonvulsant effect of WGJe (20–120 mg/kg; i.p.) in DBA/2 mice alone (blue line) and in combination with flumazenil (2.5 mg/kg; i.p.; red line). Abscissa shows the drug doses. (**A**) Ordinate shows the % of clonic seizures induced by audiogenic stimuli. (**B**) Ordinate shows the % of tonic seizures induced by audiogenic stimuli.

WGJe injection at the dose of 20 mg/kg, 30, 60 and 120 min before auditory stimulation, did not significantly (*p* > 0.05) affect the occurrence of either clonic or tonic seizures in audiogenic seizure (AGS)-prone DBA/2 mice. Conversely, the administration of WGJe from 40 to 120 mg/kg i.p., 30 min before auditory stimulation, significantly (*p* < 0.01) protected, in a dose-dependent manner, against the clonic and tonic phases of the audiogenic seizure (Figure 2A,B), with ED_50_ values of 60.68 (45.76–80.47) and 47.83 (33.17–68.96) mg/kg for clonus and tonus, respectively. Furthermore, WGJe (40–120 mg/kg) was also effective against the wild running phase of reflex seizures in DBA/2 mice, with an ED_50_ of 89.28 (62.31–127.91) mg/kg (Table 2). On the contrary, the administration of WGJe, from 40 mg/kg to 120 mg/kg, 60 and 120 min before auditory stimulation, did not significantly (*p* > 0.05) change the incidence of tonic and clonic seizures.

Interestingly, the administration of flumazenil (2.5 mg/kg, i.p.), a GABA_A_ receptor antagonist, was performed to investigate the potential contribution of this receptor to the antiseizure effects of WGJe. Flumazenil, in combination with WGJe, moved dose–response curves to the right (Figure 2A,B) and significantly (*p* < 0.01) augmented the ED_50_ values up to 98.95 (73.65–132.93) mg/kg for clonus and 73.94 (55.05–99.30) mg/kg for tonus (Table 2). Likewise, WGJe co-administered with flumazenil did not protect mice against wild running in comparison to WGJe administered alone (Table 2).

### 2.5. Open-Field Test (OFT) in Treated and Untreated DBA/2 Mice

**Figure 3:**
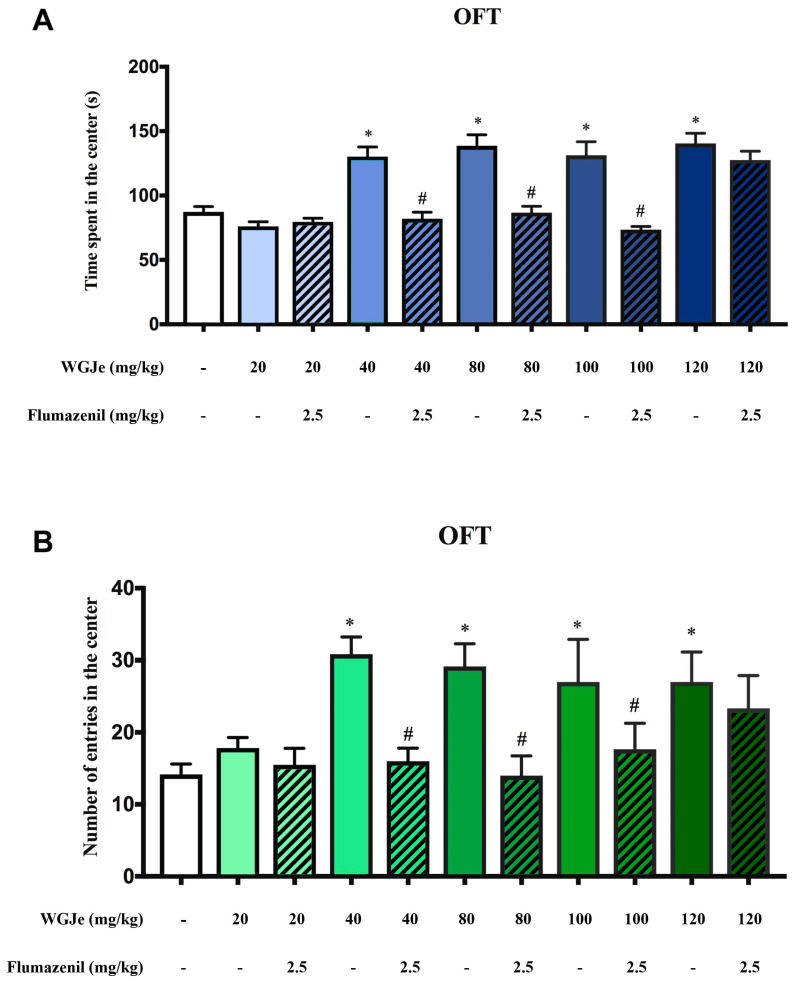
Open-field test results for anxiety measures. (**A**) Blue histograms represent the time spent in central square(s). (**B**) Green histograms represent the number of entries in the center. Dashed histograms represent co-treatment of WGJe and flumazenil. Values are expressed as means ± S.E.M. Data marked with * are significantly different (*p* < 0.01) from DBA/2-vehicle group, whereas data marked with # are significantly different (*p* < 0.01) from respective DBA/2 WGJe-treated mice.

The distance moved and mean velocity did not significantly differ between treated and untreated DBA/2 mice in the OFT, supporting the absence of a locomotor deficit. The time spent in the center along with the number of entries, which are inversely linked to the level of anxiety in mice [14], were significantly (*p* < 0.01) increased in WGJe-treated DBA/2 mice, at the doses of 40, 80, 100 and 120 mg/kg, in comparison to untreated DBA/2 mice (Figure 3A,B). The i.p. administration of flumazenil (2.5 mg/kg), 15 min before the test, was able to antagonize the anxiolytic-like effect of WGJe, at 40, 80 and 100 mg/kg, in DBA/2 mice. Conversely, the anxiolytic-like effect of WGJe at 120 mg/kg was not antagonized by flumazenil (Figure 3A,B).

### 2.6. Molecular Docking of the Main Components of WGJe in the GABA_A_ Receptor

Based on the antagonistic effects observed by the employment of flumazenil, we investigated the potential interaction between the most representative polyphenols present in WGJe and the GABA_A_ receptor. We chose the compounds present at a concentration of <2 g/kg in WGJe, excluding procyanidin B1 and B3 given their high molecular weight, while preferring their monomers. Ultimately, we assessed the binding capacity of catechin, cis-piceid, trans-coutaric acid, epicatechin, quercetin, quercetin-3-glucoronide and quercetin-3-glucoside.

**Figure 4:**
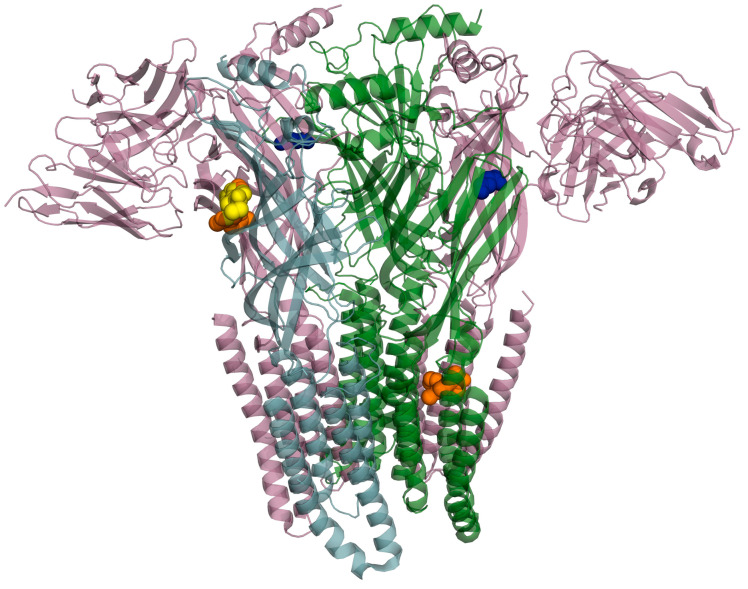
Crystallized structure of the GABA_A_ receptor bound with the endogenous ligand γ-aminobutyric acid (in blue), the agonist diazepam (in orange) and the antagonist flumazenil (in yellow; PDB: 6X3X and 6X3U superimposed).

The crystal structure of the GABA_A_ receptor bound to the positive allosteric modulator diazepam was employed (PDB: 6X3X). In detail, diazepam can bind at the classical benzodiazepine site in the extracellular domain (ECD) of the α-γ interface as well as in the transmembrane domain (TMD) at the β-α interface [15]. Therefore, these two binding domains were chosen for docking simulations of the most representative polyphenols present in WGJe (Figure 4).

As shown in Table 3, all the selected polyphenols displayed the capability to interact at both binding sites of benzodiazepines. In particular, at the ECD site, according to the ChemPLP algorithm, the compounds that scored the best were quercetin-3-glucoside (110.93) and quercetin-3-glucoronide (109.34), followed by cis-piceid (80.50), which showed also the lowest root mean square deviation (RMSD) of atomic positions, meaning it is close to the co-crystallized ligand. Interestingly, the ChemPLP scores of reference ligands diazepam and flumazenil were 77.02 and 83.96, respectively, suggesting that the best-scoring WGJe polyphenols are able to theoretically create more interactions with the GABA_A_ receptor. These results were also confirmed by the GoldScore algorithm. At the TMD site as well, the polyphenols quercetin-3-glucoside (76.39), cis-piceid (72.11) and quercetin-3-glucoronide (68.06) represented the best docked compounds, and yet the latter showed the lowest RMSD value. These values were comparable to the fitness score of diazepam docked at the same site (73.09). According to GoldScore, quercetin-3-glucoronide and quercetin-3-glucoside possessed similar fitness scores (72.72 and 72.02, respectively), followed by cis-piceid (61.13).

**Figure 5:**
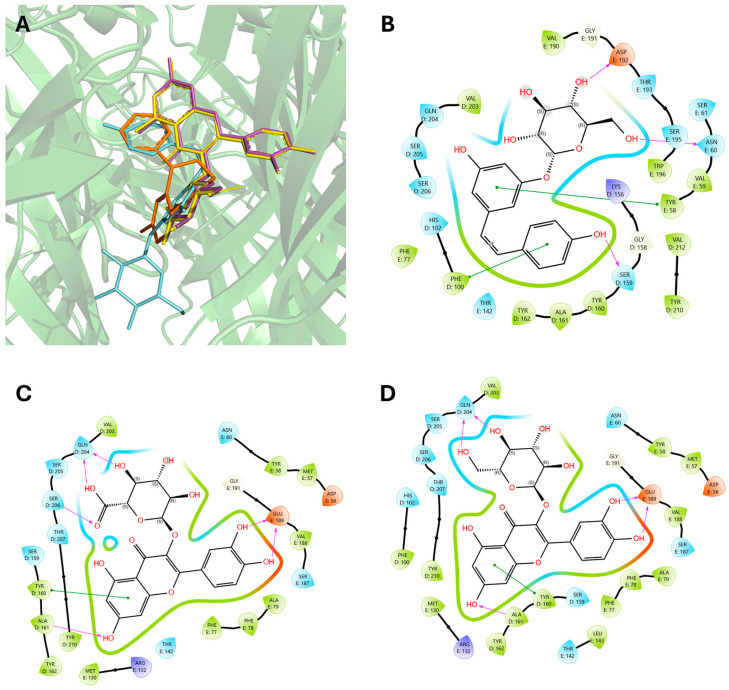
Binding modes of the highest-ranking polyphenols of WGJe and their interactions at the ECD site. (**A**) Best docked positions of quercetin-3-glucoside (in magenta), quercetin-3-glucoronide (in yellow) and cis-piceid (in cyan) compared to the crystallized ligand (diazepam, in orange) at the ECD site. (**B**–**D**) Maps of interactions between docked cis-piceid (**B**), quercetin-3-glucoronide (**C**) and quercetin-3-glucoside (**D**) at the ECD binding site. Magenta arrows represent hydrogen bonds, while green lines represent π-π stacking.

As shown in Figure 5A, the selected polyphenols followed a pattern of binding similar to that of the crystallized ligand (diazepam) at the ECD site. Regarding the highest-ranking polyphenols, cis-piceid was able to form π-π stacking with Phe100 of the α_1_ subunit, like diazepam, as well as with Tyr58 of the γ_2_ subunit. The H-bonds formed with Asn60 and Asp162 of the α_1_ subunit, along with Ser159 of the γ_2_ subunit (Figure 5B). Quercetin-3-glucoronide maintains the H-bond with Ser206, while strongly interacting with Gln240 and Glu198 of the α_1_ and γ_2_ subunits, respectively. Moreover, the benzo-γ-pyrone moiety forms π-π stacking with Tyr160 and a hydrogen bond with Ala169 of the α_1_ subunit (Figure 5C). Interestingly, the same pattern of interactions is present for quercetin-3-glucoside, though the H-bond with Ser206 is lost due to the lack of the carboxyl group in the glucose moiety (Figure 5D).

**Figure 6:**
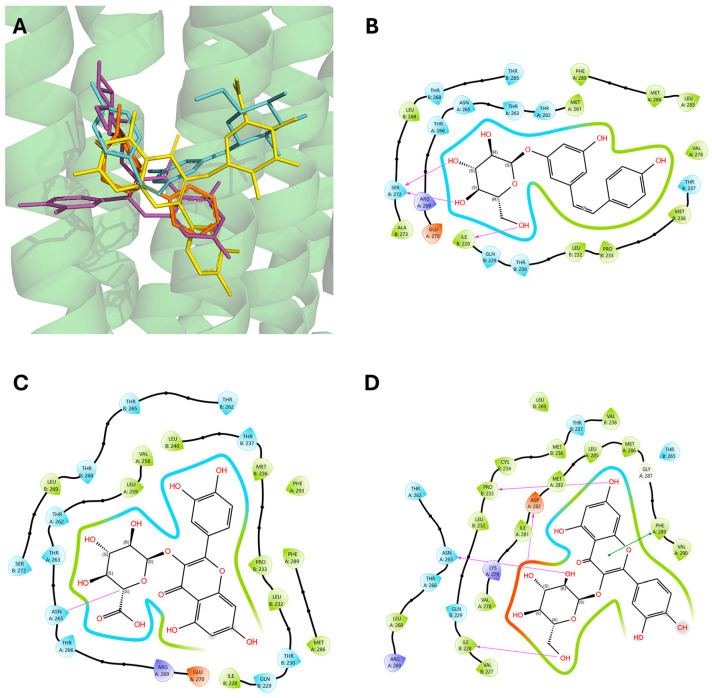
Binding modes of the highest-ranking polyphenols of WGJe and their interactions at the TMD site. (**A**) Best docked positions of quercetin-3-glucoside (in magenta), quercetin-3-glucoronide (in yellow) and cis-piceid (in cyan) compared to the crystallized ligand (diazepam, in orange) at the TMD site. (**B**–**D**) Maps of interactions between docked cis-piceid (**B**), quercetin-3-glucoronide (**C**) and quercetin-3-glucoside (**D**) at the TMD binding site. Magenta arrows represent hydrogen bonds, while amber ones stand for halogen bonds. The green lines represent π-π stacking.

Similarly to what was observed at the ECD site, WGJe polyphenols stacked at the TMD site following the binding mode of diazepam, though with differences in projection towards the core of the receptor (Figure 6A). Both quercetin-3-glucoronide (Figure 6C) and quercetin-3-glucoside (Figure 6D) form an H-bond with Asn265 of the β_2_ subunit, while the latter interacts with Ile228, like cis-piceid (Figure 6B). Interestingly, quercetin-3-glucoside also forms tight π-π stacking with Phe289 and an H-bond with Pro233, while cis-piceid interacts with Ser272.

## 3. Discussion

The scientific community has established the role of natural products in counteracting the onset of seizures. Indeed, centuries of traditional medicine have proven the capability of polyphenols, alkaloids and terpenoids to help manage epilepsy [16,17,18]. Moreover, the general population is rising quickly, particularly in developing nations, so finding affordable and easily available medications is an important goal to achieve. Additionally, since they may have various modes of action, plant-derived drugs might be of invaluable benefit in epilepsy by overcoming resistance [19,20].

Along this line, we are the first to report that WGJe possesses anti-epileptic effects, as assessed in different rodent models. In detail, we observed that WGJe was able to counteract only tonic seizures in PTZ-injected ICR-CD1 mice in a dose-dependent manner. The inefficacy of WGJe to hamper clonic seizures in PTZ-injected mice suggests that this extract may act like benzodiazepines. Gasior and co-workers demonstrated that diazepam displayed anti-epileptogenic effects against tonic seizures, and yet not on clonic ones in PTZ-injected mice [21]. Other natural matrices have shown similar effects. Indeed, our results agree with those showing that extracts of *Albizia adianthifolia* and *Phragmanthera austroarabica*, two polyphenol-rich matrices, significantly protected mice against PTZ-induced seizures, via targeting different pathways influenced by this toxin [22,23]. Interestingly, quercetin, one of the aglycones present in WGJe, was able to hinder seizures in the same in vivo model we employed, as well as prolong the onset of seizures [24].

The anti-epileptic effects we observed in PTZ-injected ICR-CD1 mice also occurred in the AGS-susceptible DBA/2 mice. Indeed, the treatment with WGJe was able to hinder the onset of epileptic outcomes, both tonic and clonic ones, in a dose-dependent manner, as well as the typical sign of wild running. We previously demonstrated that a flavonoid-rich extract of *Citrus sinensis* juice was able to hamper seizures in the same model, as well as its main flavonoids hesperidin and narirutin [25].

We also observed that in these two models, WGJe treatment produced significant results only at the shortest timing (i.e., 30 min before PTZ injection or auditory stimulus). As documented, extensive metabolism or rapid efflux from the brain may cause a loss of the anti-epileptic potential of flavonoids, as indicated for quercetin [26].

In WAG/Rij rats, a genetic model of absence epilepsy, we did not observe a significant modulation of the SWD pattern to control rats. We speculate that this might be due to the different nature of the seizures present in this model. Absence seizures are typically treated with the employment of three AEDs, such as ethosuximide, valproic acid and lamotrigine, as first-line agents [27]. Given the mode of action of these drugs, via the blockage of T-type calcium channels by ethosuximide or voltage-gated sodium channels by lamotrigine and valproic acid, the latter being slightly active also against GABA transaminase, we suggest that molecules like polyphenols, which are thought to act mainly on the GABAergic system, may not elicit significant results in an in vivo model of absence [27]. Of note, in the same experimental model, it was shown that quercetin, at a higher dose compared to what was present in WGJe, hampered absence seizures, yet by reducing proinflammatory cytokines and nitric oxide production, suggesting that other mechanisms may be involved [28].

To prove our hypothesis that WGJe might act via targeting the GABAergic system, we employed the AGS-susceptible DBA/2 mice model co-administering WGJe with flumazenil, a GABA_A_ antagonist. Indeed, we observed that flumazenil was able to abolish the effect of WGJe in DBA/2 mice in terms of both clonic and tonic seizures, as well as block the anxiolytic effects observed in the OFT, thus clearly suggesting the involvement of GABA_A_ receptors. Moreover, the highest dose of WGJe (120 mg/kg) was able to revert the antagonistic effect of flumazenil, as the polyphenolic mixture present in WGJe acted competitively to restore the anti-epileptic outcome. Others have shown that polyphenols target GABA_A_ receptors to induce their anti-epileptic effects. Indeed, resveratrol hampered kainic-acid-induced epilepsy in rats, where it reversed both chronic and silent phases of epilepsy, up-regulated the expression of the kainate glutamate receptor in the hippocampus, and down-regulated the GABA_A_ receptor along with the increased hippocampal glutamate/GABA ratio provoked by the stressor [29].

GABA_A_ receptors are ionotropic receptors coupled with a chlorine-dependent ion channel and are widespread in both the central and peripheral nervous systems. The natural agonist, GABA, allows the opening of the channel, thus leading to a rapid influx of chlorine and, consequently, reducing the membrane potential and inhibiting nervous transmission [30]. The GABA_A_ subtype is the most studied due to the wide plethora of agonists and antagonists characterized by relevant clinical implications. Moreover, GABA_A_ receptors represent one of the targets of natural compounds endowed with anti-epileptic effects [31]. Based on the effects observed after the employment of flumazenil, we wondered whether WGJe polyphenols could effectively bind to GABA_A_ receptors. To test that, we employed molecular docking, which is acknowledged to be a promising tool for the study, design and optimization of natural products [32]. Recent crystallographic studies highlighted that within the GABA_A_ receptor, there are two different binding domains: the ECD and the TMD [15,30]. Regarding the former, it has been described that diazepam forms crucial bonds with Phe100 via the benzyl group, with His102 via the chlorine atom and with Ser205 via the carbonyl group, which are relevant for its activity [30].

Our results showed that the most representative polyphenols of WGJe are able to efficiently interact with ECD. In particular, we observed that cis-piceid keeps the π-π interaction with Phe100, while the two quercetin glycosides interact with Tyr160, a residue less than 2.5 Å from Phe100, thus presumably allowing the stabilization of the subunits as allowed by the first residue to maintain an enlarged pore. This is similar to Ser205, which does not represent an interaction made by the polyphenols, whereas Gln204 and Ser206, two residues linked by peptide bonds to the crucial residue, are strongly connected with both quercetin-3-glucoside and quercetin-3-glucoronide. Remarkably, none of the polyphenols we investigated interacted with Tyr142 or with Try207, two relevant residues for the antagonistic effects of flumazenil [15].

Regarding the TMD, benzodiazepines are thought to dive into the interfaces between the two α1 and the two β2 subunits, thus creating two separate yet identical binding sites [15,30]. In these cavities, diazepam binds under Asn265 of the β subunit and Ile228 at the short π-helix within the α subunit. Moreover, the benzyl ring of diazepam makes electrostatic contacts with Asn265, which is a key residue for pre-clinical anesthetic effects [33,34,35], as well as forms stacking interactions with Phe289. Among the polyphenols we investigated, cis-piceid interacted with the relevant Ile228 while quercetin-3-glucoronide interacted with Asn265, like diazepam. Quercetin-3-glucoside shared those connections, as well as π-π interaction with Phe289 and, interestingly, it formed an H-bond with Pro233, which represents a link for the phenyl ring of diazepam to interact with both β and α subunits to maintain the GABA_A_ activated structure [15]. A novel binding site has been found for diazepam by Kim and co-workers, which lies deep between the γ and β subunits at the TMD interface. We also performed our docking simulation on this site, and yet the protocol could not be validated due to a high RMSD value for the reference ligand (> 2 Å), despite setting as constraints and keeping flexible the residues known to be involved in this interaction. This could be because the experimental conditions employed to retrieve the crystal structure are complex, and thus the genetic algorithm used by docking software cannot always provide consistent results. Previous reports have shown that natural compounds can interact with GABA_A_ subunits in the benzodiazepine sites to elicit sedative or anti-epileptic effects. Along this line, the soy isoflavone daidzein dose-dependently and significantly reduced the latency whilst increasing the sleep duration of male Swiss albino mice. This effect was counteracted by flumazenil, employed to assess the involvement of GABA_A_ receptors. Also, in this study, molecular dockings and dynamics demonstrated the binding capacity of isoflavone to interact with the benzodiazepine sites [36]. Similarly, tetrahydrolinalool, an acyclic monoterpene alcohol present in essential oils, produced anti-convulsant effects in picrotoxin-, 3-mercapto-propionic acid- and PTZ-injected mice. Moreover, the in silico studies showed that the monoterpene formed a stable complex with the GABA_A_ receptor, similar to diazepam [37].

Apart from GABA_A_, the alteration of the glutamate balance is thought to be relevant in the onset of epilepsy [38], as well as other neurotransmitters. Indeed, acetylcholine has been suggested to be involved in the excitation of neurons and epilepsy [39]. Monoamines (i.e., norepinephrine, dopamine, serotonin, histamine and melatonin) are acknowledged for their role in reducing seizures and ameliorating epileptic outcomes [40]. Another significant molecule that functions as a neurotransmitter and a second messenger is nitric oxide (NO), which is synthesized by nitric oxide synthase (NOS) [41]. This enzyme may be found either in pre- or post-synaptic neurons, and it can influence behavior, learning, memory, neurotoxicity, synaptic flexibility and neuronal signaling, thus being relevant in epilepsy [42,43]. Therefore, there are several pathways involved in this pathology, which may be influenced by WGJe in achieving anti-epileptic outcomes along with the GABAergic one.

## 4. Materials and Methods

### 4.1. Quali-Quantitative Characterization of WGJe

The WGJe used in this study was obtained from juice provided by the company “Bono & Ditta” (Campobello di Mazara, Trapani, Italy). By means of ultra-performance liquid chromatography coupled with a triple quadrupole electrospray tandem mass spectrometry (UPLC-QqQ-MS/MS), the quali-quantitative characterization of WGJe was performed. The separation of the phenolic and polyphenolic compounds was achieved at 40 °C, employing a Waters Acquity HSS T3 1.8 µm column (Milford, MA, USA). The mobile phase A consisted of a water solution of 0.1% formic acid, while the acetonitrile solution of 0.1% formic acid was mobile phase B. Through analysis, samples were kept at 4 °C and a flow rate of 0.4 mL/min. Mass spectrometry detection was accomplished, as reported [44].

### 4.2. Animals

Male WAG/Rij rats (6–7 months old, 250–300 g), DBA/2 (22–26 days old) and ICR- CD1 mice of 6 weeks of age were purchased from Charles River Laboratories Srl (Calco, Lecco, Italy). Animals were housed in groups of 10 per cage, maintaining a controlled humidity (60 ± 5%) and temperature (21 ± 2 °C), with a light cycle of 12/12 h. Food and tap water were available ad libitum until the time of the experiments. Procedures involving animals and their care were conducted in conformity with the international and national laws and policies (EU Directive 2010/63/EU for animal experiments, ARRIVE guidelines and the Basel declaration, including the 3R concept). The experimental protocol here reported was approved by the Animal Care Committee of the University of Catanzaro (Italy) and authorized by the Italian Ministry of Research and University (MIUR; authorization n° 425/2017-PR). All efforts were made to minimize animal suffering and to use only the number of animals necessary to produce reliable scientific data.

### 4.3. PTZ-Induced Seizures in ICR CD-1 Mice

This experiment protocol was performed to assess the effect of several doses of WGJe (20, 40, 80, 100 and 120 mg/kg) or its vehicle (sterile saline solution 0.9% NaCl) i.p. administered in male ICR CD-1 mice 30, 60 or 120 min before the injection of PTZ (60 mg/kg, i.p.) [45]. PTZ was purchased from Sigma Aldrich, Milan, Italy. By virtue of this, ICR CD-1 mice (n = 180) were randomly divided into 6 groups (per dose and vehicle), and each of these into 3 subgroups, 1 for the different times of administration (before the injection of PTZ). Ten mice were present in each subgroup. Briefly, mice were placed in a 30 × 30 × 30 cm Plexiglas box and monitored for 30 min. During this observational time, seizures were scored as previously described [46]. Clonic spasms were considered significant if they lasted more than 5 s each. Absence of this threshold convulsion over 30 min revealed that the mouse was protected from seizures [47]. Times and routes of drug administration were chosen based on previously published papers or personal pilot studies [25,47,48].

### 4.4. Audiogenic Seizures in DBA/2 Mice

To investigate the effects of WGJe treatment on DBA/2 mice, we carried out two experimental protocols. The experiments on DBA/2 mice, weighing 8–12 g (22–26 days old), were performed according to the method previously described [47].

#### 4.4.1. Experimental Protocol #1

DBA/2 mice were exposed to auditory stimulation 30, 60 and 120 min following i.p. administration of WGJe (20, 40, 80, 100 and 120 mg/kg) or a vehicle [25,47]. To perform this, DBA/2 mice (n = 180) were randomly divided into 6 groups (n = 30 per dose and vehicle) and each of these into 3 subgroups (n = 10) for the different times of administration before auditory stimulation. Briefly, each mouse, used only once, was sited under a hemispheric Perspex dome (diameter 58 cm), and the auditory stimulation (12–16 kHz, 109 dB) was delivered as previously described, as well as further scoring [25]. The maximum response was recorded for each mouse. Behavioral changes were monitored during the time of drug administration and auditory stimulation.

#### 4.4.2. Experimental Protocol #2 (Co-Administration Protocol)

To investigate the contribution of the GABA_A_ receptor to the anti-seizure effects of WGJe, flumazenil (Hoffmann-LaRoche, Basel, Switzerland), at a dose of 2.5 mg/Kg, was i.p. administered 15 min after the injection of WGJe at several doses (20, 40, 80, 100 and 120 mg/kg), whereas the auditory stimulus was delivered 30 min after WGJe injection, as previously described [25]. Times and routes of flumazenil administration were chosen based on previously published papers in which it was shown not to worsen reflex seizures when administered alone. The total number of DBA/2 mice that developed audiogenic seizures was scored at each dose used [47,49].

### 4.5. Experiments in WAG/Rij Rats

To investigate the role of WGJe in counteracting absence seizures, male WAG/Rij rats were used as a well-validated model of absence epilepsy and epileptogenesis with neuropsychiatric comorbidities. For EEG recordings, WAG/Rij rats of ~6 months of age, under anesthesia, were stereotaxically implanted with 3 cortical electrodes for EEG recordings, as previously described [50]. After surgery, all WAG/Rij rats were allowed to rest for at least 1 week of recovery. For at least 72 h before the experiments, animals were connected to the recording cables to habituate them to the recording conditions. Following this period, rats were connected to a multichannel amplifier (Pinnacle Technology’s 8400–9000 video/EEG system with Sirenia Software 1.7.9, Lawrence, KS, USA) by a flexible recording cable and an electric swivel, sited above the cages, allowing free movements for the animals [50]. WAG/Rij rats (n = 8 for each dose) were i.p. administered with WGJe (20, 40, 80, 100 and 120 mg/kg) or saline. The number and duration of SWDs was assessed by video-EEG recording, as described [50]. All EEG signals were amplified and conditioned, and they were digitally converted with a sampling rate of 300 Hz. The quantification of absence seizures was performed as previously described [51].

### 4.6. Behavioral Test

Different groups of mice, for each dose used, were examined in the OFT, in order to study the effects of acute WGJe treatment, administered 30 min before the test, on the development of anxiety-related behavior in DBA/2 mice (n = 10 per vehicle and dose). Furthermore, other groups of mice (n = 10 per dose of WGJe and vehicle) also received flumazenil at 2.5 mg/kg i.p., in order to investigate the potential contribution of the GABA_A_ receptor to the anxiolytic-like effect of WGJe.

This test was performed under a controlled temperature and humidity, whereas light intensity was determined by the experimental setup. Each behavioral test started at 9:00 am and finished before 11:00 am to avoid potential circadian alteration of the test results [47].

#### Open-Field Test (OFT)

Anxiety-like behavior was monitored for 10 min in an open field arena, a white 70 × 70 cm Plexiglas apparatus with the floor divided into 9 squares. Each mouse was gently placed in the central square of the apparatus and its activity scored. Before each trial, the arena was systematically cleaned. During the test, the time spent in the center and the number of center entries were analyzed, for both treated and untreated mice, in order to test anxiety-like behavior. Lower exploratory activity in the OFT is usually taken as a measure of an increased level of anxiety and vice versa. Moreover, the total distance moved (cm), and the mean velocity (sec) during the 10 min test were also statistically analyzed, for treated and untreated mice, in order to test locomotor activity.

### 4.7. Statistical Analysis

All data are given as the mean ± SEM. The statistical software used was GraphPad Prism 6.0 (La Jolla, CA, USA). Regarding DBA/2 mice, statistical comparisons among groups were carried out through Fisher’s exact probability test (incidence of the seizure phases). The percent occurrence of audiogenic seizure phases was evaluated for each administered compound, and the dose–response curves were close-fitted through the linear regression method, as previously described [47]. ED_50_ values (±95% confidence limits) for each compound and each seizure phase were calculated as reported [25]. The scores of seizure severity were compared among groups by employing the Kruskall–Wallis nonparametric analysis of variance (ANOVA), which was followed by a Mann–Whitney U-test. Regarding WAG/Rij rats, the duration and number of SWDs were evaluated separately for every 30 min epoch [25,47]. Data was analyzed by one-way ANOVA, followed by Bonferroni’s post hoc test. All tests were two-sided, with *p* < 0.05 considered significant.

### 4.8. Docking Studies

The ligand structures of the chosen WGJe polyphenols were designed using the Maestro 14.0 software (Schrödinger LLC; New York, NY, USA) and minimized to produce low-energy 3D structures in order to further proceed with the docking studies. We set a pH range of 7.2–7.4 to mimic physiological conditions. The docking studies were performed by GOLD software (Hermes 2024.1.0, Cambridge Crystallographic Data Centre; Cambridge, UK). The crystal structure of the GABA_A_ receptor in complex with diazepam was retrieved from the RCSB Protein Data Bank (entry code: 6X3X) [15]. The ligands and water molecules were removed, while hydrogens were added by Maestro 14.0 software (Schrödinger LLC). Docking simulations were performed, setting the region of interest to contain the residues within 10 Å from the position of diazepam in the X-ray structure. GoldScore and ChemPLP were employed as fitness functions. Default settings were used, and the ligands were submitted to 100 genetic algorithm runs. The results differing by less than 0.75 Å in ligands—all atom RMSDs were clustered together. The interactions of the best-ranked conformations with the surrounding residues were investigated by Maestro 14.0 software (Schrödinger LLC). Images were acquired by PyMOL 2.6.0 software (Schrödinger LLC) [52].

## 5. Conclusions

Our results demonstrate that WGJe employed in this study hampered the onset of seizures in PTZ-injected ICR CD-1 mice and in AGS-sensible DBA/2 mice. The antagonistic effect of flumazenil suggests that GABA_A_ receptors are involved in the anti-epileptic and anxiolytic effects of WGJe. Indeed, docking simulations supported the ability of the most abundant polyphenols present in WGJe to interact with the benzodiazepine sites of GABA_A_ receptor, unravelling their putative mode of action. Given the multi-target capability of natural products and the complexity of epilepsy, other mechanisms of action may contribute to the seizure-inhibiting effects exerted by WGJe. Altogether, this extract may represent an efficient tool for epilepsy management.

## Figures and Tables

**Table 1:** Quali-quantitative composition of WGJe performed by UPLC/QqQ–MS/MS analysis. Results are expressed as mg/kg of dry extract.

Class	Compounds	Concentration (mg/kg)
Flavonols	Quercetin-3-glucuronide	15,531.7
	Quercetin-3-glucoside	6470.2
	Quercetin-3,4-rutinoside	1266.1
	Kaempferol-3-glucuronide	828.2
	Kaempferol-3-glucoside	409.5
	Isorhamnetin-3-glucoside	359.5
	Quercetin-3-glucoside-arabinoside	115.1
	Rutin	34.2
	Quercetin	27.5
	Kaempferol-3-rutinoside	4.1
	Quercetin-3-glucoside acetyl	4.1
	Isorhamnetin-3-rutinoside	3.8
Flavanols	Procyanidin B1	7326.9
	Catechin	3355.8
	Procyanidin B3	2086.2
	Epicatechin	379.1
Hydroxycinnamates	trans-Coutaric acid	2740.2
	Caffeic acid	371.5
	Chlorogenic acid	33.6
	p-Coumaric acid	23.5
	Ferulic acid	21.1
Resveratrols	cis-Piceid	2295.7
	trans-Piceid	57.7
Phenolic acids	Ellagic acid	867.6
	p-hydroxybenzoic acid	85.4
	Vanillic acid	64.3
	2,6-diOH-benzoic acid	5.1
	Methyl gallate	2.1
Dihydroflavonols	Taxifolin	491.2
	Dihydrokaempferol	60.6
Dihydrochalcones	Phlorizin	40.1
	Trilobatin	20.3
Flavanones	Hesperidin	21.1
Flavones	Luteolin-7-O-glucoside	6.3
	Luteolin	0.8
	Sinensetin	0.5
Hydroquinones	Arbutin	295.2

**Table 2:** ED_50_ values of PTZ-injected ICR-CD1 mice treated with WGJe, and ED_50_ values of audiogenic seizures in DBA/2 mice treated with WGJe alone or co-administered with flumazenil (2.5 mg/kg), after 30 min from drug administration. All data reported are expressed as mg/kg and were extrapolated following the Litchfield and Wilcoxon method (1949). Values in parentheses are 95% of the confidence limit. * *p* < 0.01, statistically significant differences from the vehicle-drug control group. **^#^** *p* < 0.01, statistically significant differences from the WGJe-treated group.

Treatment	Dose Range (mg/kg)	Mice Model	Seizure Phase		
			Wild Running	Clonus	Tonus
WGJe	20–120	PTZ treated ICR-CD1 mice	/	NE	57.76 (45.48–73.36) *
WGJe	20–120	DBA/2 mice	89.28 (62.31–127.91) *	60.68 (45.76–80.47) *	47.83 (33.17–68.96) *
WGJe + Flumazenil (2.5 mg/kg)	20–120	DBA/2 mice	NE	98.95 (73.65–132.93) ^#^	73.94 (55.05–99.30) ^#^

NE: not effective.

**Table 3:** WGJe polyphenols ranked by their docking score. The table shows the 2D structure of each polyphenol, as well as the corresponding numerical output from the GoldScore and ChemPLP algorithms.

Compound	Structure	Docking Score			
		ECD		TMD	
		GoldScore	ChemPLP	GoldScore	ChemPLP
Catechin	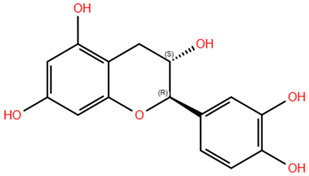	64.67	75.51	56.69	66.14
Cis-piceid	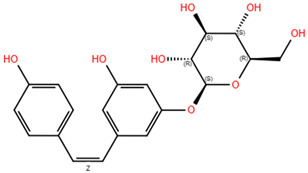	72.03	80.50	61.13	72.11
Coutaric acid	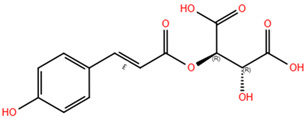	64.04	75.40	51.55	62.14
Epicatechin	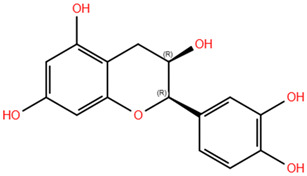	62.92	78.21	56.11	66.75
Quercetin	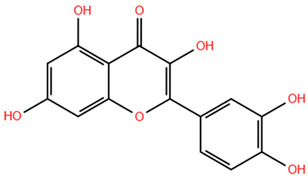	66.11	75.86	53.56	60.24
Quercetin-3-glucoronide	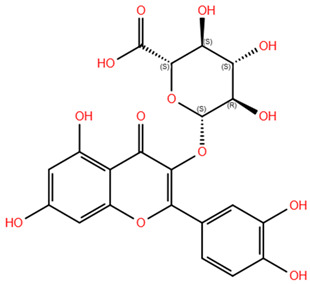	75.57	109.34	72.72	68.06
Quercetin-3-glucoside	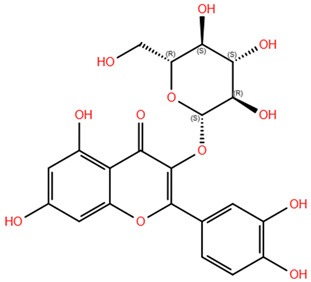	80.14	110.93	72.02	76.39

## Data Availability

The original contributions presented in this study are included in the article. Further inquiries can be directed at the corresponding author.
